# Factors Associated with Preoperative Anxiety in Patients Undergoing Ambulatory Hand Surgery: A Cross-Sectional Observational Study

**DOI:** 10.3390/jcm13237004

**Published:** 2024-11-21

**Authors:** Justyna Napora, Krystian Gryglewski, Miłosz Piotrowicz, Piotr Lebiedź, Tomasz Mazurek, Katarzyna Nowicka-Sauer

**Affiliations:** 1 Department of Orthopaedics and Traumatology, Faculty of Medicine, Medical University of Gdańsk, 80-803 Gdańsk, Poland; 2 Institute of Applied Mathematics, Faculty of Applied Physics and Mathematics, Gdańsk University of Technology, 80-233 Gdańsk, Poland; 3 Department of Family Medicine, Faculty of Medicine, Medical University of Gdańsk, 80-211 Gdańsk, Poland; 4 Department of Cardiac Surgery, Kashubian Centre for Cardiac and Vascular Diseases, Ceynowa Specialist Hospital, 84-200 Wejherowo, Poland

**Keywords:** hand surgery, carpal tunnel syndrome, day surgery, perioperative care, preoperative anxiety, rehabilitation in hand surgery, prehabilitation, APAIS

## Abstract

**Background:** Studies examining preoperative anxiety in patients awaiting hand surgery are scarce. Preoperative anxiety is a common reaction and can have a negative impact on treatment outcomes. The aim of this study was to assess the level of anxiety in patients undergoing hand surgery as a one-day procedure and to investigate the associations between patients’ preoperative anxiety and selected sociodemographic, psychological, and clinical variables. **Methods:** We examined 121 patients (77.7% women) who were operated on in an ambulatory setting. The mean age was 52.6 years (range: 24–84 years). Preoperative anxiety was assessed according to the Amsterdam Preoperative Anxiety and Information Scale (APAIS). The Visual Analogue Scale was used to assess irritability, depression, and pain. **Results:** Univariate analyses showed significant correlations between patients’ preoperative anxiety and increased age, surgery within a year since diagnosis, the presence of rehabilitation in their medical history, higher irritability, and living in rural areas. Multivariate analyses showed significant associations between patients’ anxiety level and diagnosis of up to a year, a history of rehabilitation and the level of irritability. **Conclusions:** Patients undergoing hand surgery in an ambulatory surgery setting experience some preoperative anxiety. Younger patients, those with a shorter duration of disease, with a history of rehabilitation, those presenting intense irritability, and those living in rural areas may demand special attention.

## 1. Introduction

Although day surgery has been practised for years, increased demand for elective procedures and other socio-economic factors have led to the dominance of day surgery settings for elective procedures [1]. Over 50% of elective procedures in Europe and in the United States are day surgeries. An ambulatory procedure is a non-emergency procedure [2].

The ambulatory setting is preferred over in-patient surgery for most hand and wrist surgeries [3]. Outpatient surgery takes place independently of the standard operation of the hospital unit. Ambulatory hand surgery is cost-effective, safe, and related to higher patient comfort [4]. One-day surgery is less disruptive to patients and has a lower incidence of hospital-acquired infections. However, it has been shown that shorter periods of perioperative care may exacerbate anxiety [5]. Anxiety is among the most common psychological reactions in the preoperative period, with a prevalence of nearly 80%. The prevalence of anxiety in day-care surgery may reach up to 40% [6].

Hand conditions treated operatively in day surgery settings include carpal tunnel syndrome (CTS), tendovaginitis, Dupuytren’s contracture, cubital tunnel syndrome, and de Quervain syndrome, among others. Heightened anxiety is often reported among patients with CTS and other upper extremity conditions [7,8,9]. Carpal tunnel syndrome is a common diagnosis. It mainly affects middle-aged patients, with a significant female predominance [10]. There is a suggested association between preoperatively assessed patient-reported symptom severity, electrodiagnostic severity, problematic initial recovery, and return to full-duty work [11,12,13,14]. Anxiety symptoms are also a predictor of more severe pain at follow-up after hand-related disease treatment [15,16]. Psychological distress related to musculoskeletal diseases and complaints has been proven to be related to long-term disability [8,9,15]. Heightened preoperative anxiety is associated with poorer outcomes such as higher consumption of analgesic medication, prolonged wound healing, a higher complication rate, higher risk of early readmission, prolonged hospital stay and recovery, lower patient satisfaction with treatment, lower motivation for rehabilitation, kinesiophobia, long-term pain, and increased risk of disability, morbidity, and mortality, including for patients undergoing various types of surgery, including orthopedic procedures [17,18,19,20,21,22,23,24,25]. For arthroplasty, preoperative anxiety is also a suggested risk factor for early readmission [20,21,24]. Preoperative anxiety seems to be a multifactorial phenomenon; thus, exploring its determinants in a particular population would allow for more personalized and effective management [26].

Recent studies revealed that both occupational therapists and surgeons are more focused on the biomedical approach. As a consequence, psychological factors can be marginalized or even ignored [8,27]. The holistic approach of health professionals engaged in treatment and rehabilitation is more beneficial for outcomes. Bidirectional association between chronic pain and anxiety and depression, and their impact on patient engagement, adherence, and motivation, justify incorporating assessment of psychological state in recommendations [28,29,30,31].

In light of the abovementioned significant associations between preoperative anxiety and poorer outcomes of various types of surgery, including orthopedics, it is recommended that routine assessments of psychological status, including anxiety, be conducted both preoperatively (during prehabilitation) and postoperatively [8,9,27].

The aim of our study was to assess the preoperative anxiety level in patients undergoing hand surgery in the ambulatory setting and to examine associations between patients’ anxiety and specific sociodemographic, clinical, and psychological factors. This study may contribute to the knowledge of the modifiable (e.g., depression, irritability, analgesic intake) and non-modifiable (e.g., sociodemographic factors, diagnosis, handedness, previous surgeries, and rehabilitation) factors associated with anxiety. An understanding of these factors may facilitate the preparation of effective interventions in the prehabilitation process by encouraging practitioners to focus on at-risk groups. We hypothesize that women, younger patients, and those who are professionally active may experience more severe anxiety. We also suppose that less severe anxiety would be experienced by patients who have undergone previous surgeries, since past experiences of surgery decrease the sense of “not knowing” about the process of hospitalization and surgical treatment and increase the patient’s sense of control. We also hypothesize that those who are scheduled for a non-dominant hand operation may experience less anxiety. It seems that dominant hand surgery may cause concern related to more difficulties in daily functioning or possible delays in the patient’s return to work. We also speculate that previous rehabilitation may be associated with a higher level of anxiety related to surgery. We also suppose that a longer time from diagnosis may be related to less anxiety. The latter may be at least partially explained by the adaptive process. In addition, we hypothesize that patients with more severe anxiety may declare a higher level of enduring negative emotions, namely depression and irritability.

## 2. Materials and Methods

This cross-sectional observational study was approved by the Independent Bioethics Committee for Scientific Research. The study report was prepared according to STROBE (Strengthening the Reporting of Observational Studies in Epidemiology) recommendations. All patients gave written informed consent to participate in the study. The study was conducted over a 7-month period between September 2019 and February 2020.

Inclusion criteria included planned hand surgery in a one-day setting, age 18 years and above, informed consent, and the ability to complete the questionnaire independently. Criteria for exclusion were age under 18 years, hospitalization for longer than one day, inability to complete the questionnaire independently, and refusal to participate in the study. To address the potential study bias, all consecutive patients meeting inclusion criteria were enrolled in the study. The minimum sample size calculated for the study was 114 (confidence interval 95%, margin error 5%, and population proportion 50%).

Our study included 121 patients (77.7% women). All patients were operated on under intravenous regional anesthesia. The mean age was 52.6 years (SD = 12.4; range: 24–84 years). On the day of surgery, clinical and sociodemographic data were collected and patients self-assessed their preoperative anxiety using the Amsterdam Preoperative Anxiety and Information Scale (APAIS). APAIS is a standardized method created specifically to assess the level of anxiety among patients awaiting surgical procedures. It is a six-item questionnaire, including four items used to measure preoperative anxiety (two concern anxiety related to anesthesia and two concern anxiety related to surgery). The remaining two items assess information requirements regarding anesthesia and surgery. Patients assess the level of their anxiety/information desire on a 5-point Likert scale (1—“not at all” to 5—“extremely”). The maximum score for the anxiety scale is 20 points [25,32]. The APAIS is widely used in surgical studies, including orthopedics and hand surgery [33,34,35,36,37]. The analysis of the need for information scale will be the focus of a separate study. The Polish version of the APAIS used in this study was validated among patients scheduled for cardiac surgery. The psychometric properties (validity and reliability) of the Polish version of the APAIS are satisfactory [38]. The levels of irritability, depression, and pain were measured using the Visual Analogue Scale (VAS), with possible scores ranging from 0 (“none”) to 10 points, with the latter indicating the highest level of the studied variables. A structured interview was used to gather clinical and sociodemographic data.

## 3. Statistical Analysis

Results are presented with means with standard deviations and medians and minimal and maximum values. Numbers and percentages are presented for categorical variables. The statistical analysis aimed at identifying the predictors of patient preoperative anxiety in a univariate and multivariate way. Taking into consideration the distribution of preoperative anxiety, we decided to use a negative binomial regression, which is a general linear model (GLM) based on the negative binomial (Pascal) distribution. Multivariate analyses were conducted using a backward elimination method until all parameters that remained in the model were statistically significant (*p*-value ≤ 0.05). Statistical analysis was performed with R (R core team, 2021).

## 4. Results

**Figure 1:**
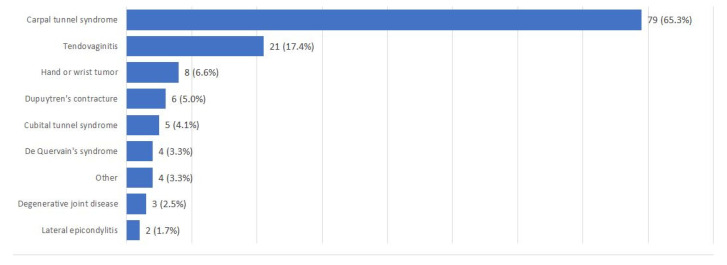
Distribution of hand diseases.

One hundred and twenty-one patients were included in the study. The patients’ characteristics are presented in Table 1 and Figure 1.

Seventy-nine participants (65.3%) were operated on for carpal tunnel syndrome. Eleven patients (9.1%) were operated on for multiple conditions within one visit. The distribution of diseases is presented in Figure 1.

A total of 57.9% of patients were operated on for a condition affecting the right hand. The dominant hand was involved in 57.9% of patients. A total of 40.5% of patients had a diagnosis of a disease with a duration of less than one year. The procedure was a second procedure or surgical revision for 21.5% of patients. A total of 65.3% of patients had undergone some kind of surgical procedure before, and 14.9% of the patients had taken analgesics daily (Table 1).

Among 121 included patients, the mean APAIS anxiety score was 9.3 (SD = 3.7) with a median of 9 (range: 4–20 points). The mean APAIS anxiety score was 9.6 points for women and 8.1 points for men (Table 2).

Pain and psychological variables were assessed using VAS (possible range: 0–10 points). The results are presented in Table 3.

Univariate analysis demonstrated significant correlations between preoperative anxiety and age (age was inversely proportional with APAIS score; *p* = 0.039). Patients operated on within one year since their diagnosis had significantly higher APAIS scores (*p* = 0.006); patients who underwent rehabilitation had higher APAIS scores (*p* = 0.011); higher VAS irritability was associated with a higher APAIS score (*p* = 0.006); patients from rural areas presented with a higher APAIS anxiety score (*p* = 0.042). Multivariate analyses showed that a time from diagnosis shorter than one year (*p* < 0.001), rehabilitation in history (*p* < 0.001), and higher VAS irritability score (*p* = 0.003) were statistically significantly associated with higher preoperative anxiety (Table 4).

## 5. Discussion

The goal of our study was to assess preoperative anxiety and the factors associated with its occurrence in patients undergoing hand surgery in a one-day surgery setting. According to the available literature, preoperative anxiety can adversely affect patients’ psychological and physiological states [39,40,41]. Patients’ emotional reactions, in turn, can affect therapeutic outcomes [42,43].

In our study, the mean score of preoperative anxiety was 9.3 points. The observed level of anxiety was quite high given the one-day surgery setting. A review of the literature revealed a small number of studies that used the APAIS to investigate preoperative anxiety within patients scheduled for orthopedic surgery. In comparison, in one prospective study on hand surgery, the level of preoperative anxiety according to the APAIS was 8 points before interventions to reduce anxiety were taken [36]. In another study, the mean level of preoperative anxiety on the APAIS in patients undergoing surgery for a fracture of the distal end of the radius under WALANT anesthesia was 7.78 points. In contrast, in a group of patients undergoing surgery for the same indication but under general anesthesia, the mean level of anxiety on the APAIS was 7.36 [37]. De Caro et al. showed that the level of preoperative anxiety on the APAIS, in a population of people with hip or knee osteoarthritis was 9.1 points [44]. In a study by Raghavan et al., the mean baseline preoperative anxiety level on the APAIS in patients awaiting total knee or hip replacement was approximately 8 points [45]. It is worth emphasizing that the level of anxiety among hand surgery patients in our study was similarly high compared to the level of anxiety in patients scheduled for hip and knee surgery. Thus, routine assessment of anxiety among hand surgery candidates seems vital. In our study, the mean level of preoperative anxiety on the APAIS was 9.6 among women and 8.1 among men. This result concurs with our hypothesis and other authors’ observations [22,26,32,38,43,46].

As expected, our results indicate that preoperative anxiety decreases as the patient’s age increases. These results may also be related to the higher levels of anxiety observed in professionally active participants. Lower levels of anxiety, as assessed by the APAIS in older patients undergoing hand surgery, were also reported by Touil et al. [36]. In a study by Jia et al., 15.5% of patients over 50 years of age diagnosed with cubital canal syndrome (CuTS) exhibited anxiety, while among those aged between 18 and 50 years, 10.8% of patients showed anxiety [47]. However, these results did not show statistical significance. The presence of anxiety was assessed in the cited study using the Hospital Anxiety and Depression Scale (HADS). Tasdemir et al. observed lower levels of preoperative anxiety in patients aged 51–70 than in patients aged 18–50 years [48]. However, this result was not found to be statistically significant. Anxiety severity was assessed by the state–trait anxiety inventory (STAI) scale. It should be noted that the differences between the results of our study and those quoted may be mainly due to the study’s methodology. Both HADS and STAI assess general levels of anxiety, whereas only APAIS refers directly to preoperative anxiety. According to our hypothesis, patients with a time since diagnosis of less than a year had significantly higher levels of anxiety than patients with a longer time since diagnosis. There have been few studies within orthopedics examining the relationship between time since diagnosis and anxiety levels. Jia et al. reported that the proportion of patients presenting anxiety was similar in groups of patients diagnosed with CTS more than 2 years and 2 years or less before surgery (14.5% vs. 13.9%). This finding did not show statistical significance [47]. Janzen and Hadjistarvropoulos, in a study in which 82% of participants awaited an orthopedic procedure and 18% were waiting to receive a general procedure, showed that participants were more fearful about waiting for surgery than the surgery itself [49].

Our study revealed, contrary to our hypothesis, a lack of significant association between previous surgeries and preoperative anxiety. However, interestingly, the current study revealed that patients who underwent rehabilitation in the past showed significantly higher anxiety compared to those who did not undergo such treatment. One possible hypothesis to explain this result could be that patients who underwent rehabilitation may hope that it would be effective enough to avoid surgery. However, this hypothesis requires verification in further studies. Inverse results were observed by Brand et al. and Kunikata et al. In the cited studies, a lower anxiety level was observed after patients received a hand massage right before the surgery [39,40]. It seems to be an important point that hand massage can cause an increase in parasympathetic nervous system tone and a decrease in sympathetic nervous system tone, manifesting as a slowed heart rate and other consistent findings [40]. Given that we do not have data on the type of rehabilitation undergone by our patients, it is difficult to comment on these results. It should also be mentioned that prehabilitation, including physiotherapy, has been proven to be effective in surgical patients in terms of various outcomes, including patients scheduled for total shoulder replacement [50,51,52,53]. Future studies on prehabilitation in hand surgery would shed more light on this issue. An analysis of the correlation between the anxiety of patients undergoing outpatient hand surgery procedures with a history of rehabilitation may be a rewarding area for further research.

We hypothesized that more intense preoperative anxiety would correlate with more severe depression and irritability. Our predictions are partially consistent with the results which showed that it was irritability rather than depression that was strongly associated with anxiety. There are studies demonstrating that irritability often correlates with anxiety in both clinical and environmental studies [54,55,56,57,58]. Surgery is a stressful factor with many possible emotional reactions, e.g., anxiety, lowered mood, and tension, but also anger and irritability [46,57]. A previous study on patients’ fears before surgery for carpal tunnel syndrome in an outpatient setting found that both irritability and anxiety (as measured by the VAS scale) were elevated in patients who feared postoperative pain. Stecz and Kocur observed that anger was significantly associated with anxiety in patients undergoing hip replacement [42,59]. It is worth mentioning that anger may be one of the risk factors for postoperative pain after hip and knee arthroplasty [60]. Irritability was also one of the correlates of suicidal ideation in patients who had undergone cardiac surgery [61]. Thus, it seems that anger and irritability may serve as significant emotional reactions in surgical patients; this theory is worthy of additional research and psychological preoperative interventions [57]. Since we did not find studies presenting a correlation between irritability and anxiety before outpatient hand surgery procedures, we believe that this is an interesting field for future studies. Such a suggestion seems even stronger in the light of results of a multivariate analysis which revealed that a higher level of irritability is a significant factor in terms of high anxiety, along with time from diagnosis and previous rehabilitation.

Current results indicate that higher anxiety occurs in patients living in rural areas. A similar association was observed by Kuzminskaitė et al. among patients undergoing non-cardiac surgery, but this finding lacked statistical confirmation [22]. Contradictory results were shown in a study conducted at Gondar University Hospital, examining a population of adults scheduled for elective surgery [62]. The absence of any correlation between the place of residence (urban vs. rural) was shown by a study conducted in Ethiopia, on a population consisting of adult patients scheduled for elective surgery [63]. However, the results of these studies should be approached with caution, as the cultural and economic context of villages in African countries differs significantly from that in Europe. Each position on the matter has its own socio-behavioural theories to explain the correlations between the place of residence and anxiety levels. In light of the heterogeneous findings on limited populations that cannot be easily compared, this issue is worthy of further exploration.

Contrary to our hypothesis, our study showed no difference in preoperative anxiety between patients awaiting surgery on their dominant hand and those awaiting surgery on their non-dominant hand. In a study conducted by Tessler et al., it was shown that patients undergoing revision amputation on the dominant hand showed higher anxiety compared to patients undergoing amputation on the non-dominant hand [64]. Kang et al. presented that patients with trapeziometacarpal arthritis of the dominant hand had higher expectations regarding treatment than patients with trapeziometacarpal arthritis of the non-dominant hand [65]. Our results suggest that hand surgery “itself” can be a significant source of preoperative anxiety, irrespective of the fact whether the dominant or non-dominant hand requires surgery.

Our study has important clinical implications, since it allowed us to identify factors strongly associated with high anxiety levels among patients scheduled for daytime hand surgery. The results indicate that patients who were diagnosed during the year before the surgery, have undergone rehabilitation, and experience or present symptoms of irritation or anger may require special attention from health professionals, including psychological support.

## 6. Conclusions

The results of this study highlight the importance of preoperative anxiety in patients undergoing outpatient hand surgery procedures. Our study showed that patients undergoing hand surgery in the day-surgery setting experience some level of preoperative anxiety. Higher preoperative anxiety was observed among patients who were diagnosed at least one year before the surgery in comparison with those who were diagnosed earlier. More intense anxiety was also related to a history of rehabilitation. Among psychological factors, more severe anxiety was associated with more intense irritability. And last but not least, younger patients and those living in rural areas were more anxious. The association between prior rehabilitation and higher anxiety levels seems especially interesting. However, it demands further research. Outpatient hand surgery is usually less traumatic than most other surgical and orthopedic procedures. Nevertheless, it is also associated with increased levels of preoperative anxiety and should be a matter of further research. Studies exploring negative emotions experienced by patients prior to hand surgery, including irritability, and interventions tailored to increase patients’ emotional well-being are vital.

## 7. Limitations

A strong limitation of the study was the large sex disproportion of the participants (77.7% were women). However, it is worth mentioning that patients were operated on for various diseases, with most patients (65.3%) being operated on for carpal tunnel syndrome. According to sources, this condition is predominant among women. The uneven distribution of diseases makes it impossible to compare results according to diagnosis. All patients included in the study were operated on under intravenous retrograde anesthesia, which made it impossible to compare anxiety related to this and other anesthesia techniques. The cross-sectional, exploratory character of the study excludes cause-and-effect conclusions. The lack of follow-up assessment prevented us from observing the impact of preoperative anxiety on surgery outcomes. Such a study is underway as the next stage of the current project. We believe that prospective evaluation would shed more light on the described associations. The study was conducted in a single centre. Thus, generalizations should be made with caution.

## Figures and Tables

**Table 1:** Sociodemographic and clinical characteristics of the studied sample (*n* = 121).

Characteristic	*n* (%) *
Age [years; mean (SD)]	52.6 (12.4)
Sex	
Women	94 (77.7)
Men	27 (22.3)
Education	
Primary	6 (5.0)
Vocational	22 (18.2)
Secondary	59 (48.8)
University	34 (28.1)
Employment status	
Retired or receiving disability pension	45 (37.2)
Employed	76 (62.8)
Physical work	44 (36.4)
Mental work	32 (26.4)
Residence	
Alone	20 (16.5)
With family	101 (83.5)
Marital status	
Married or have a partner	98 (81.0)
Single	23 (19.0)
Place of residence	
City	94 (77.7)
Countryside	27 (22.3)
Affected Hand	
Right	70 (57.9)
Left	51 (42.1)
Handedness	
Right	117 (96.7)
Left	4 (3.3)
Time since diagnosis	
<1 year	49 (40.5)
1–2 years	19 (15.7)
2–5 years	26 (21.5)
More than 5 years	27 (22.3)
History of any surgery	
Yes	79 (65.3)
No	42 (34.7)
Contralateral hand procedure	
Yes	26 (21.5)
No	95 (78.5)
Rehabilitation in history	
Yes	27 (22.3)
No	94 (77.7)

* Data presented as number and percentage, unless otherwise stated.

**Table 2:** Level of preoperative anxiety on the APAIS with differentiation by sex (*n* = 121).

Sex	Mean (SD)	Median (Min–Max)
Women (*n* = 94)	9.6 (3.6)	9 (4–20)
Men (*n* = 27)	8.1 (3.8)	7 (4–17)

**Table 3:** The level of pain, irritability, and depression in the studied sample (*n* = 121).

Variable *	Mean (SD)	Median (Min–Max)
Pain	6.0 (2.5)	7 (0–10)
Irritability	4.7 (2.9)	5 (0–10)
Depression	4.0 (2.6)	4 (0–10)

* Assessed with the use of the Visual Analogue Scale (VAS) with a possible range of 0–10 points.

**Table 4:** Results of the analysis of factors associated with preoperative anxiety.

**Univariate Analysis**	***p*** **-Value**
Diagnosis until one year	0.000 *
Irritability (VAS)	0.006 *
Rehabilitation in history	0.011 *
Age	0.039 *
Living in the countryside	0.042 *
Sex	0.054
Carpal tunnel syndrome	0.080
Depression (VAS)	0.131
Pain (VAS)	0.155
Primary education	0.206
History of any surgery	0.271
Living with family	0.535
Unemployment	0.631
Daily analgesics intake	0.636
In a relationship	0.727
Mental work	0.795
Dominant hand	0.826
Contralateral hand procedure	0.945
**Multivariate Analysis**	***p*** **-Value**
Diagnosis until one year	0.000 *
Irritability (VAS)	0.006 *
Rehabilitation in history	0.011 *

* Statistically significant.

## Data Availability

The data that support the findings of the study are available on request from the corresponding author.

## References

[1] Pereira L., Figueiredo-Braga M., Carvalho I.P. (2016). Preoperative anxiety in ambulatory surgery: The impact of an empathic patient centered approach on psychological and clinical outcomes. Patient Educ. Couns..

[2] De Lathouwer C., Poullier J.P. (2000). How much ambulatory surgery in the World in 1996-1997 and trends?. Ambul. Surg..

[3] Thompson N.B., Calandruccio J.H. (2018). Hand surgery in the ambulatory surgery center. Orthop. Clin. N. Am..

[4] Goyal K.S., Jain S., Buterbaugh G.A., Imbriglia J.E. (2016). The safety of hand and upper-extremity surgical procedures at a freestanding ambulatory surgery center. J. Bone Jt. Surg..

[5] McIntosh S., Adams J. (2011). Anxiety and quality of recovery in day surgery: A questionnaire study using Hospital Anxiety and Depression Scale and Quality of Recovery Score. Int. J. Nurs. Pract..

[6] Wetsch W.A., Pircher I., Lederer W., Kinzl J.F., Traweger C., Heinz-Erian P., Benzer A. (2009). Preoperative stress and anxiety in day-care patients and inpatients undergoing fast-track surgery. Br. J. Anaesth..

[7] Beleckas C.M., Wright M., Prather H., Chamberlain A., Guattery J., Calfee R.P. (2018). Relative prevalence of anxiety and depression in patients with upper extremity conditions. J. Hand Surg. Am..

[8] Gil J., Goodman A.D., Mulcahey M.K. (2018). Psychological factors affecting outcomes after elective shoulder surgery. J. Am. Acad. Orthop. Surg..

[9] Belayneh R., Haglin J., Lott A., Kugelman D., Konda S., Egol K.A. (2019). Underlying mental illness and psychosocial factors are predictors of poor outcomes after proximal humerus repair. J. Orthop. Trauma.

[10] Aroori S., Spence R.A. (2008). Carpal tunnel syndrome. Ulster Med. J..

[11] Cowan J., Makanji H., Mudgal C., Jupiter J., Ring D. (2012). Determinants of return to work after carpal tunnel release. J. Hand Surg..

[12] Jerosch-Herold C., Houghton J., Blake J., Shaikh A., Wilson E.C.F., Shepstone L. (2017). Association of psychological distress, quality of life and costs with carpal tunnel syndrome severity: A cross-sectional analysis of the PALMS cohort. BMJ Open.

[13] Ryan C., Miner H., Ramachandran S., Ring D., Fatehi A. (2022). General anxiety is associated with problematic initial recovery after carpal tunnel release. Clin. Orthop. Relat. Res..

[14] Shin Y.H., Yoon J.O., Kim Y.K., Kim J.K. (2018). Psychological status is associated with symptom severity in patients with carpal tunnel syndrome. J. Hand Surg. Am..

[15] Degen R.M., MacDermid J.C., Grewal R., Drosdowech D.S., Faber K.J., Athwal G.S. (2016). Prevalence of symptoms of depression, anxiety, and posttraumatic stress disorder in workers with upper extremity complaints. J. Orthop. Sports Phys. Ther..

[16] Egloff N., Wegmann B., Juon B., Stauber S., Von Känel R., Vögelin E. (2017). The impact of anxiety and depressive symptoms on chronic pain in conservatively and operatively treated hand surgery patients. J. Pain Res..

[17] Bedaso A., Mekonnen N., Duko B. (2022). Prevalence and factors associated with preoperative anxiety among patients undergoing surgery in low-income and middle-income countries: A systematic review and meta-analysis. BMJ Open.

[18] Cserep Z., Losoncz E., Balog P., Szili-Török T., Husz A., Juhász B., Kertai M.D., Gál J., Székely A. (2012). The impact of preoperative anxiety and education level on long-term mortality after cardiac surgery. J. Cardiothorac. Surg..

[19] Hanusch B.C., O’Connor D.B., Ions P., Scott A., Gregg P.J. (2014). Effects of psychological distress and perceptions of illness on recovery from total knee replacement. Bone Jt. J..

[20] Jackson K.L., Rumley J., Griffith M., Agochukwu U., DeVine J. (2020). Correlating psychological comorbidities and outcomes after spine surgery. Glob. Spine J..

[21] Jiménez Ortiz M., Espinosa Ruiz A., Martínez Delgado C., Barrena Sánchez P., Salido Valle J.A. (2020). Do preoperative anxiety and depression influence the outcome of knee arthroplasty?. Reumatol. Clin..

[22] Kuzminskaitė V., Kaklauskaitė J., Petkevičiūtė J. (2019). Incidence and features of preoperative anxiety in patients undergoing elective non-cardiac surgery. Acta Med. Litu..

[23] Munafò M.R., Stevenson J. (2001). Anxiety and surgical recovery. J. Psychosom. Res..

[24] Nixon D.C., Schafer K.A., Cusworth B., McCormick J.J., Johnson J., Klein S.E. (2019). Preoperative anxiety effect on patient-reported outcomes following foot and ankle surgery. Foot Ankle Int..

[25] Zemła A.J., Nowicka-Sauer K., Jarmoszewicz K., Wera K., Batkiewicz S., Pietrzykowska M. (2019). Measures of preoperative anxiety. Anaesthesiol. Intensive Ther..

[26] Celik F., Edipoglu I.S. (2018). Evaluation of preoperative anxiety and fear of anesthesia using APAIS score. Eur. J. Med. Res..

[27] Kurrus M.B., Jewell V.D., Gerardi S., Gerg M., Qi Y. (2023). Psychosocial factors addressed by occupational therapists in hand therapy: A mixed methods study. J. Hand Ther..

[28] McGrath R.L., Shephard S., Parnell T., Verdon S., Pope R. (2024). Recommended approaches to assessing and managing physiotherapy clients experiencing psychological distress: A systematic mapping review. Physiother. Theory Pract..

[29] Chiesa M., Nicolini G., Buoli M. (2024). The Approach of Physiotherapists in the Management of Patients with Persistent Pain and Comorbid Anxiety/Depression: Are There Any Differences between Male and Female Professionals?. Medicina.

[30] Silva Guerrero A.V., Maujean A., Campbell L., Sterling M. (2018). A Systematic Review and Meta-Analysis of the Effectiveness of Psychological Interventions Delivered by Physiotherapists on Pain, Disability and Psychological Outcomes in Musculoskeletal Pain Conditions. Clin. J. Pain.

[31] Jack K., McLean S.M., Moffett J.K., Gardiner E. (2010). Barriers to treatment adherence in physiotherapy outpatient clinics: A systematic review. Man. Ther..

[32] Moerman N., van Dam F.S.A.M., Muller M.J., Oosting H. (1996). The Amsterdam Preoperative Anxiety and Information Scale (APAIS). Anesth. Analg..

[33] Ossai E.N., Nwosu A.D.G., Onwuasoigwe O., Ubboe K., Ameh J., Alu L. (2023). Prevalence and predictors of anxiety among surgical patients in the preoperative holding area of National Orthopaedic Hospital, Enugu, Nigeria: A Cross-Sectional Study. J. West Afr. Coll. Surg..

[34] Meunier V., Mares O., Gricourt Y., Simon N., Kouyoumdjian P., Cuvillon P. (2022). Patient satisfaction after distal upper limb surgery under WALANT versus axillary block: A propensity-matched comparative cohort study. Hand Surg. Rehabil..

[35] Sertcakacilar G., Yildiz G.O., Bayram B., Pektas Y., Cukurova Z., Hergunsel G.O. (2022). Comparing preoperative anxiety effects of brachial plexus block and general anesthesia for orthopedic upper-extremity surgery: A randomized, controlled trial. Medicina.

[36] Touil N., Pavlopoulou A., Momeni M., Van Pee B., Barbier O., Sermeus L., Roelants F. (2021). Evaluation of virtual reality combining music and a hypnosis session to reduce anxiety before hand surgery under axillary plexus block: A prospective study. Int. J. Clin. Pract..

[37] Abd Hamid M.H., Abdullah S., Ahmad A.A., Singh P.S.G.N., Soh E.Z.F., Liu C.Y., Sapuan J. (2021). A randomized controlled trial comparing Wide-Awake Local Anesthesia with No Tourniquet (WALANT) to general anesthesia in plating of distal radius fractures with pain and anxiety level perception. Cureus.

[38] Nowicka-Sauer K., Banaszkiewicz D., Jarmoszewicz K., Hajduk A., Stefaniak J., Janiszewska J., Beta S., Siebert J. (2018). Validation of the Amsterdam Preoperative Anxiety and Information Scale among patients scheduled for cardiac surgery. J. Cardiovasc. Surg..

[39] Brand L.R., Munroe D.J., Gavin J. (2013). The effect of hand massage on preoperative anxiety in ambulatory surgery patients. AORN J..

[40] Kunikata H., Watanabe K., Miyoshi M., Tanioka T. (2012). The effects measurement of hand massage by the autonomic activity and psychological indicators. J. Med. Investig..

[41] Ni C.H., Wei L., Wu C.C., Lin C.H., Chou P.Y., Chuang Y.H., Kao C.C. (2021). Machine-Based hand massage ameliorates preoperative anxiety in patients awaiting ambulatory surgery. J. Nurs. Res..

[42] Napora J., Gryglewsk K., Piotrowicz M., Nowicka-Sauer K., Mazurek T. (2021). Analysis of concerns among patients operated for carpal tunnel syndrome. Chir. Narzadow. Ruchu. Ortop. Pol..

[43] Salzmann S., Rienmüller S., Kampmann S., Euteneuer F., Rüsch D. (2021). Preoperative anxiety and its association with patients’ desire for support—An observational study in adults. BMC Anesthesiol..

[44] De Caro M.F., Vicenti G., Abate A., Picca G., Leoncini V., Lomuscio M., Casalino A., Solarino G., Moretti B. (2015). Optimal improvement in function after total hip and knee replacement: How deep do you know your patient’s mind?. J. Biol. Regul. Homeost. Agents.

[45] Raghavan G., Shyam V., Murdoch J.A.C. (2019). A survey of anesthetic preference and preoperative anxiety in hip and knee arthroplasty patients: The utility of the outpatient preoperative anesthesia appointment. J. Anesth..

[46] Caumo W., Schmidt A.P., Schneider C.N., Bergmann J., Iwamoto C.W., Bandeira D., Ferreira M.B.C. (2001). Risk factors for preoperative anxiety in adults. Acta Anaesthesiol. Scand..

[47] Jia S., Shi X., Liu G., Wang L., Zhang X., Ma X., Li J., Shao X. (2020). Determinants of anxiety and depression in patients with cubital tunnel syndrome. BMC Psychiatry.

[48] Tasdemir A., Erakgün A., Nuri Deniz M., Çertuǧ A. (2013). Comparison of preoperative and postoperative anxiety levels with state-trait anxiety inventory test in preoperatively informed patients. Turk. J. Anaesthesiol. Reanim..

[49] Janzen J.A., Hadjistavropoulos H.D. (2008). Examination of negative affective responses to waiting for surgery. Can. J. Nurs. Res..

[50] Banasiewicz T., Kobiela J., Cwaliński J., Spychalski P., Przybylska P., Kornacka K., Bogdanowska-Charkiewicz D., Leyk-Kolańczak M., Borejsza-Wysocki M., Batycka-Stachnik D. (2023). Rekomendacje w zakresie stosowania prehabilitacji czyli kompleksowego przygotowania pacjenta do zabiegu operacyjnego. Pol. Przegl. Chir..

[51] López Rodríguez-Arias F., Sánchez-Guillén L., Armañanzas Ruiz L.I., Lara C.D., Gómez F.J.L., Pons C.B., Rodríguez J.M.R., Arroyo A. (2020). A Narrative review about prehabilitation in surgery: Current situation and future perspectives. Cir. Esp. (Engl. Ed.).

[52] Tsimopoulou I., Pasquali S., Howard R., Desai A., Gourevitch D., Tolosa I., Vohra R. (2015). Psychological prehabilitation before cancer surgery: A systematic review. Ann. Surg. Oncol..

[53] Villers J.F., Burch J., Scheller M., Huang H.H. (2020). Physical therapy prehabilitation on a reverse total shoulder replacement candidate: A case study. J. Phys. Ther. Sci..

[54] Cornacchio D., Crum K.I., Coxe S., Pincus D.B., Comer J.S. (2016). Irritability and anxiety severity among youth with anxiety. J. Am. Acad. Child Adolesc. Psychiatry.

[55] Crum K.I., Hwang S., Blair K.S., Aloi J.M., Meffert H., White S.F., Tyler P.M., Leibenluft E., Pope K., Blair R.J.R. (2021). Interaction of irritability and anxiety on emotional responding and emotion regulation: A functional MRI study. Psychol. Med..

[56] Kircanski K., White L.K., Tseng W.L., Wiggins J.L., Frank H.R., Sequeira S., Zhang S., Abend R., Towbin K.E., Stringaris A. (2018). A latent variable approach to differentiating neural mechanisms of irritability and anxiety in youth. JAMA Psychiatry.

[57] Levett D.Z.H., Grimmett C. (2019). Psychological factors, prehabilitation and surgical outcomes: Evidence and future directions. Anaesthesia.

[58] Stoddard J., Stringaris A., Brotman M.A., Montville D., Pine D.S., Leibenluft E. (2014). Irritability in child and adolescent anxiety disorders. Depress. Anxiety.

[59] Stecz P., Kocur J. (2014). Coping with anxiety in patients undergoing hip replacement. Pol. J. Appl. Psychol..

[60] Wei S., Li L., Yang X., Li X., Jiang Q. (2020). Psychological interventions in the pain management after hip and knee arthroplasty: A mini review. Ann. Jt..

[61] Jarmoszewicz K., Topolski M., Hajduk A., Banaszkiewicz D., Nowicka-Sauer K. (2022). Prevalence and predictors of suicidal ideation in patients following cardiac surgery. World J. Surg..

[62] Ferede Y.A., Bizuneh Y.B., Workie M.M., Admass B.A. (2022). Prevalence and associated factors of preoperative anxiety among obstetric patients who underwent cesarean section: A cross-sectional study. Ann. Med. Surg..

[63] Bedaso A., Ayalew M. (2019). Preoperative anxiety among adult patients undergoing elective surgery: A prospective survey at a general hospital in Ethiopia. Patient Saf. Surg..

[64] Tessler O., Bartow M.J., Tremblay-Champagne M.P., Lin A.M., Landes G., Sebbag S., Nikolis A. (2017). Long-term health-related quality of life outcomes in digital replantation versus revision amputation. J. Reconstr. Microsurg..

[65] Kang L., Nguyen J., Hashmi S.Z., Lee S.K., Weiland A.J., Mancuso C.A. (2017). What demographic and clinical characteristics correlate with expectations with trapeziometacarpal arthritis?. Clin. Orthop. Relat. Res..

